# Comparing Ebola virus disease and antimicrobial resistance outbreaks in Nigeria- a cross sectional survey of awareness level of health care workers and members of community

**DOI:** 10.1186/2047-2994-4-S1-P1

**Published:** 2015-06-16

**Authors:** I Yusuf, AH Arzai, M Yushau, L Garba

**Affiliations:** 1 Microbiology (Biotechnology and Bio-molecular Sciences), Universiti Putra Malaysia, Selangor, Kuala Lumpur, Malaysia; 2 Microbiology, Bayero University Kano, Nigeria, Kano, Nigeria

## Introduction

Ebola virus disease (EVD) outbreak in Nigeria has raised the level of awareness of both health care workers (HCWs) and members of community (MCs) on the threat posed by infectious diseases and need for improvement on infection control practices, but awareness of dangers of increasing antimicrobial resistance (AMR) remained low.

## Objectives

To compare awareness level of HCWs and MCs on dangers of EVD and AMR and their control and to give educational intervention on dangers of AMR and its control to MCs with no prior knowledge of AMR.

## Methods

A cross-sectional survey of 195 HCWs and 265 MCs was conducted through structured questionnaire and interview.

## Results

Majority of HCWs (95.4%) and MCs (82.8%) have recent knowledge of EVD dangers and give reasons like EVD way of killing, stigmatization, no drugs and vaccines as reason for their awareness. Only 17.2% of MCs were aware of AMR as problem, and only 3.4% of MCs and 10.3% of HCWs agreed that AMR is more deadly than EVD. However, 76.4% Drs, 95.1% nurses, 67.9% lab scientist, 66.7% pharmacists, 77.4% students and 100% of civil servants, drivers and religious leaders believed that EVD is more horrific and spread faster, while in reality its dangers is a drop in the ocean when compared with AMR. They both attributed the rapid awareness of EVD in Nigeria despite being new, to seriousness with which stakeholders and media fight EVD, the gesture AMR is yet to receive. All agreed that prevention, not treatment is the best option to tackle EVD. About 84.1% of HCWs and only 17.2% MCs believed that careful use of antibiotics can reduce cases of AMR. After short briefing of some MCs on dangers of AMR and its control through prudent use of antibiotics, a shift in belief from 17.2% to 48.7% was observed, with 64.8% civil servants, 54.1% students, 18.2% drivers 25.5% villagers and 25.0% traditional healers changed their method of control option to prevention rather than treatment.

## Conclusion

Despite extreme panic over EVD, awareness on dangers of AMR and its control remained very low among MCs. Efforts put in place during EVD outbreak by all stakeholders and the media need to be double to increase the knowledge of both HCWs and MCs toward AMR.

## Disclosure of interest

None declared.

